# Reduced caloric intake and periodic fasting independently contribute to metabolic effects of caloric restriction

**DOI:** 10.1111/acel.13138

**Published:** 2020-03-11

**Authors:** Nikkhil Velingkaar, Volha Mezhnina, Allan Poe, Kuldeep Makwana, Richa Tulsian, Roman V. Kondratov

**Affiliations:** ^1^ Department of Biological, Geological, and Environmental Sciences and Center for Gene Regulation in Health and Disease Cleveland State University Cleveland OH USA; ^2^ Institute of Genetics and Cytology National Academy of Science of Belarus Minsk Belarus

**Keywords:** caloric restriction, circadian rhythms, fasting, gene expression, glucose homeostasis, insulin sensitivity, longevity, metabolism

## Abstract

Caloric restriction (CR) has positive effects on health and longevity. CR in mammals implements time‐restricted (TR) feeding, a short period of feeding followed by prolonged fasting. Periodic fasting, in the form of TR or mealtime, improves metabolism without reduction in caloric intake. In order to understand the relative contribution of reduced food intake and periodic fasting to the health benefits of CR, we compared physiological and metabolic changes induced by CR and TR (without reduced food intake) in mice. CR significantly reduced blood glucose and insulin around the clock, improved glucose tolerance, and increased insulin sensitivity (IS). TR reduced blood insulin and increased insulin sensitivity, but in contrast to CR, TR did not improve glucose homeostasis. Liver expression of circadian clock genes was affected by both diets while the mRNA expression of glucose metabolism genes was significantly induced by CR, and not by TR, which is in agreement with the minor effect of TR on glucose metabolism. Thus, periodic fasting contributes to some metabolic benefits of CR, but TR is metabolically different from CR. This difference might contribute to differential effects of CR and TR on longevity.

## Graphical abstract

Periodic fasting does not affect blood glucose and glucose sensitivity, but has modest effect on glucose metabolism genes expression. Periodic fasting, on the other hand, reduces blood insulin and partially improves insulin sensitivity, thus indicating that some of the metabolic benefits observed on CR are due to periodic fasting. Partial improvement in insulin sensitivity under periodic fasting may provide mechanistic explanation for modest increase in lifespan on mealtime feeding.

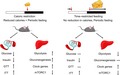

## 1 INTRODUCTION

Caloric restriction (CR), a universal lifespan increasing diet, affects many physiological systems, improves glucose and fat homeostasis, reduces level of insulin, and increases insulin sensitivity (Fontana & Klein, 2007; Masoro, McCarter, Katz, & McMahan, 1992). CR delays aging and reduces the incidence of many diseases including cardiovascular diseases, metabolic disorders, and cancer (Anderson & Weindruch, 2007). Mechanistically, CR affects several signaling pathways known to be associated with aging and metabolism: IGF, mTOR, AMPK, and sirtuin signaling (Balasubramanian, Howell, & Anderson, 2017).

Rodents are the most popular model to study caloric restriction in mammals (Mitchell et al., 2016). There are several ways to implement CR to rodents. One common method of food delivery is when a reduced amount of food (about 60%–80% of daily intake) is provided as a single meal once per day, usually, at the same time of the day. This type of CR induces strong food anticipation, and animals usually consume the food in a short (1–3 hr) period of time following a 21‐hr period of fasting (Acosta‐Rodríguez, Groot, Rijo‐Ferreira, Green, & Takahashi, 2017; Bruss, Khambatta, Ruby, Aggarwal, & Hellerstein, 2010; Mitchell et al., 2019; Patel, Chaudhari, Chaudhari, Gupta, Velingkaar, & Kondratov, 2016). Thus, CR is a self‐imposed time‐restricted (TR) feeding. TR feeding, when an unlimited amount of food is provided for a limited time frame, significantly improves metabolic health of mice on high‐fat (HF) or high‐sugar diets, and this improvement in metabolism has been linked with restored or increased circadian rhythms in gene expression and signaling (Chaix, Zarrinpar, Miu, & Panda, 2014; Hatori et al., 2012; Sherman et al., 2012). Importantly, most TR studies were conducted in context of high‐fat diet, obesity, or circadian rhythm disruption (Chaix et al., 2014; Hatori et al., 2012; Sherman et al., 2012). Much less is known about the effect of TR on regular chow in healthy mammals. Mealtime feeding (MTF) is another example of TR diet: 100% of daily food is provided once per day as a single meal; for unknown reasons, animals consume all food during a limited time frame in about 8–12 hr (Khapre et al., 2014; Mitchell et al., 2019). Importantly, MTF increases longevity in mice independent of the caloric intake, suggesting that manipulation with the feeding schedule might have beneficial effects on longevity; however, the effect on lifespan is not as strong as the effect of CR (Mitchell et al., 2019). All three interventions: CR, TR, and MTF are periodic feeding/fasting diets. It was hypothesized that fasting can improve metabolism. Indeed, fasting mimicking diets such as ketogenic diet and intermittent fasting have positive effects on metabolism and, in some cases, on longevity which supports the potential importance of periodic fasting in health (Anson et al., 2003; Roberts et al., 2017).

Feeding/fasting cycle is intertwined with circadian rhythms (Bass & Lazar, 2016). Circadian rhythms are 24‐hr rhythms in behavior, physiology, and metabolism generated by the circadian clock, a time keeping system that helps organisms synchronize internal physiological processes with their periodic environment (Eckel‐Mahan & Sassone‐Corsi, 2013). The mammalian circadian system is organized as a network of circadian clocks operating in almost every organ and synchronized by the central oscillator in the suprachiasmatic nucleus of the hypothalamus. Circadian clocks are entrained by different external cues, and light is the most important one; however, feeding also affects circadian rhythms (Longo & Panda, 2016). The circadian clock operates as a molecular feedback loop formed by several transcription factors that regulate the expression and activity of one another (Eckel‐Mahan & Sassone‐Corsi, 2013). The importance of the clock and rhythms in physiology is supported by multiple reports of negative effects of clock disruption on health (Bass & Lazar, 2016). Dietary interventions such as CR, HF, or ketogenic diet affect the circadian clock and rhythms. CR increases the amplitude of clock gene expression and reprograms circadian rhythms in transcription, translation, hormone secretion, and mTOR signaling (Makwana, Gosai, Poe, & Kondratov, 2018; Patel, Velingkaar, Velingkaar, Makwana, Chaudhari, & Kondratov, 2016; Sato et al., 2017; Tulsian, Velingkaar, & Kondratov, 2018). In turn, many metabolic benefits of caloric restriction, including effects on lifespan, are impaired in circadian clock mutants indicating an interaction between CR and the circadian clock (Katewa et al., 2016; Patel, Chaudhari, et al., 2016). In contrast to the effects of CR, high‐fat diet is known to have multiple negative effects on health and metabolism and disrupts circadian rhythms (Kohsaka et al., 2007). These rhythms can be restored by TR feeding, which correlates with improvement in metabolism (Hatori et al., 2012; Sherman et al., 2012).

This complex interaction between feeding/fasting, the circadian clocks, metabolism, and longevity brings up an important question: Are some beneficial effects of CR due to periodic prolonged fasting? To answer this question, we compared the effect of ad libitum (AL), CR, and TR on physiology and metabolism in mice. We investigated circadian rhythms in glucose homeostasis, blood glucose, insulin levels, liver gene expression, and mTORC1 signaling in mice subjected to these three diets for 2 months. We found that the 12‐hr periodic fasting contributes to some metabolic changes induced by CR such as reduced blood insulin and increased insulin sensitivity but not to other CR effects such as improved glucose homeostasis. Thus, our data support the importance of both the reduced caloric intake and temporal component of CR and suggest some mechanistic explanation on how MT or TR might affect longevity and why the effect of CR on lifespan is stronger.

## 2 RESULTS AND DISCUSSION

### 2.1 TR did not change body weight and daily food intake

**Figure 1:**
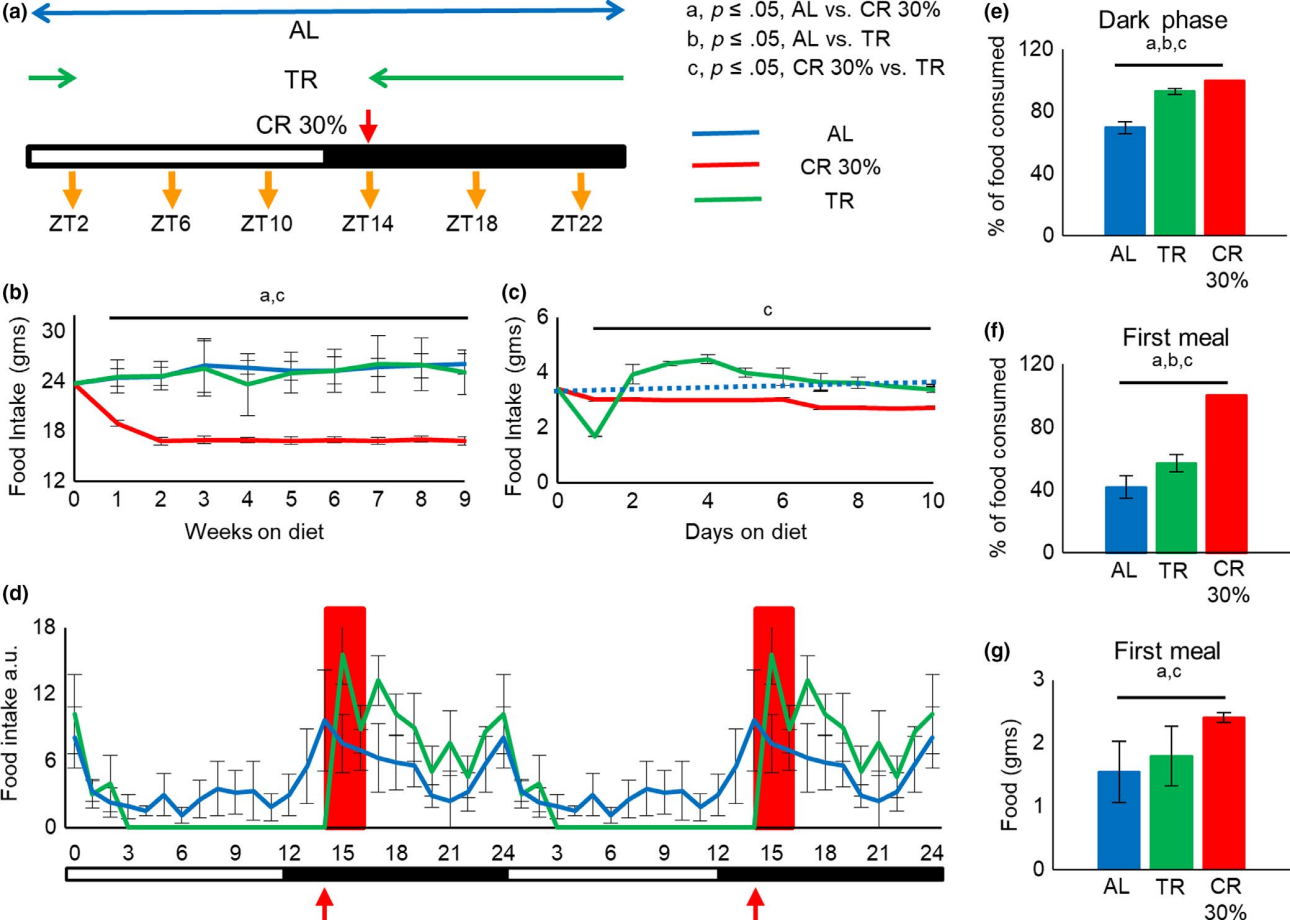
TR did not change daily pattern of food intake and body weight. (a) Scheme of feeding protocol. Mice were randomly assigned into three groups: Ad libitum feed (AL) had unlimited access to the food around the clock, 30% caloric restriction (CR 30%), and time‐restricted feed (TR) for 8–10 weeks on the respective diet, at the start of the experiments. Analysis was performed around the clock at times indicated by yellow arrows. (b) Weekly average food intake (AL (*n* = 8 cages), CR (*n* = 8 cages), and TR (*n* = 6 cages)) and (c) daily average food intake (AL (*n* = 8 cages), CR (*n* = 8 cages), and TR (*n* = 6 cages)). Daily average food intake for AL group for the first ten days was not measured; only weekly measurements were performed; and hence, AL is represented by dotted lines. For first 10 days food intake (c): AL—blue dotted line, blue triangles; TR—green solid line, green solid diamonds; CR—red solid line, red squares; for (b): AL—blue solid line, blue triangles; TR—green solid line, green solid diamonds; CR—red solid line, red squares. (d) Hourly food intake for mice on different diets. The data were normalized to daily food intake. The total daily food intake was set up as 1.0. AL—blue solid line, TR—green solid line, CR—red box. Data were double plotted for illustrative purposes. AL (*n* = 7 cages), CR (*n* = 6 cages), and TR (*n* = 5 cages). (e) Percent of daily food intake during the dark phase for mice on different diets. AL (*n* = 7 cages), CR (*n* = 6 cages), and TR (*n* = 5 cages). (f) Percent of daily food intake during the first meal. AL (*n* = 7 cages), CR (*n* = 6 cages), and TR (*n* = 5 cages). (g) Amount of food consumed as first meal. AL (*n* = 7 cages), CR (*n* = 6 cages), and TR (*n* = 5 cages). For (e‐f) AL—blue bars, TR—green bars, and CR—red bars. The time of the day when the food was provided for CR and TR mice is indicated by the red arrow. All data represented as Mean ± *SD*. One‐way ANOVA with Bonferroni correction for multiple comparison was performed. Letters indicate significant effect of the diet (*p* < .05); a—AL versus CR, b—AL versus TR, c—CR versus TR. Light was turned on at ZT0, and light was turned off at ZT12. Light and dark bars indicate light and dark phases of the day

The study design is presented on Figure 1a. Before the start of the experiment, all mice were on AL. The food intake at the start of the intervention was 3.4 ± 0.3 g. Mice on AL diet continued with unlimited access to the food. Mice on CR diet received 70% of their daily food intake as a single meal once per day at ZT14, 2 hours after the light is turned off. Mice on 12‐hr TR received an unlimited amount of food between ZT14 and ZT2. The time of feeding for CR and TR groups was selected based on normal feeding pattern of AL mice (see Acosta‐Rodríguez et al. (2017) and Figure 1d). Food intake for all three diets was monitored once every week (Figure 1b). No significant difference in food intake was observed between AL and TR mice through the duration of the study. CR group received a fixed amount: 10% restriction for first 7 days, followed by 20% restriction for next 7 days and 30% restriction for the rest of the experiment. The food consumption for TR mice was monitored every day during the first 10 days (Figure 1c). TR mice consumed about 60% less food on day 1 (Figure 1c), and the reduced consumption was most likely because AL mice eat around the clock (see Figure 1d). The food intake significantly increased on days 2–4; however, after day 4, the food intake was similar between TR and AL groups. Thus, mice on TR diet learn in a couple days that food will be provided during a limited period of time. The food intake was not measured for AL mice every day for first ten days, we did not expect any difference in food intake for these mice, and the food intake was fixed for CR mice.

During the first ten days, we monitored body weight for mice on all three diets every day, and then, weights were measured once per week during the rest of the experiment. Relative changes in body weight, normalized to every mouse body weight at the start of the experiment and absolute body weight of individual mice, are presented in Figures S1 and S2. There was a significant reduction in body weight in the CR group and no significant difference in body weight between AL and TR, similar to Acosta‐Rodríguez et al. (2017) study. Therefore, the effect of TR on normal chow is different from HF diet and future studies must be aimed at investigating the interaction between TR and diet composition.

### 2.2 Daily patterns of food intake for mice on AL, CR, and TR diets

AL mice have unlimited access to an unlimited amount of food, TR mice have limited access to an unlimited food, and CR mice have unlimited access to a limited amount of food. The restrictions might influence the daily pattern of food intake which, in turn, might affect the metabolism. The patterns of food intake for mice on all three diets are presented in Figure 1d. In agreement with previously published data (Acosta‐Rodríguez et al., 2017; Ellacott, Morton, Woods, Tso, & Schwartz, 2010), AL mice consumed about 70% of their daily food intake during the dark phase and about 30% during the light phase of the day (Figure 1e). During the dark phase, AL mice consumed the food as two major meals. The first meal was between ZT13 and ZT18, and mice consumed approximately 40% (about 1.54 g) of their daily food intake. The second large meal was between ZT22 and ZT24. Mice on TR are entrained to eat their food within a 12‐hr period, and they consumed about 90% of their food during the dark phase in two large meals. The first meal was between ZT14 and ZT17, and it was about 60% (about 1.79 g) of daily food intake, and the second large meal was between ZT22 and ZT24. CR mice consumed all 100% (about 2.4 g) of provided food between ZT14 and ZT16 (Figure 1f and g), which is in agreement with previously published studies (Acosta‐Rodríguez et al., 2017; Mitchell et al., 2019). Thus, mice on all three diets have their 1st major meal at the same time of the day.

### 2.3 Effect of diet on the rhythms in circadian clock gene expression

There is more and more evidence that circadian rhythms in peripheral organs are influenced by diet, and these rhythms can be damped, enhanced, or phase‐shifted (Hatori et al., 2012; Kohsaka et al., 2007; Patel, Velingkaar, et al., 2016). When the food for CR animals is provided around ZT12‐ZT14, CR affects the amplitude but not the phase of circadian clock gene expression in the liver (Patel, Velingkaar, et al., 2016). TR feeding affects the amplitude of clock gene expression for mice on HF diet and affects the phase of expression if food is restricted during the light phase (Damiola et al., 2000; Hatori et al., 2012).

We assayed mRNA expression for several circadian clock genes. The daily rhythms in mRNAs for *Bmal1, Per1, Per2, and Rev‐erbα* genes in the liver of mice on AL, CR, and TR diets are shown in Figure S3a‐d, and circadian rhythmicity of genes expression analyzed using JTK‐Cycle is shown in Table S3. CR did not change the phase of clock gene expression in agreement with previous reports, and TR caused about 2‐hr phase delay for *Bmal1, Per1, and Rev‐erbα* and no phase shift for *Per2.* Little to no effect on phases of clock gene expression correlated with feeding patterns suggesting that diets did not disrupt the core clock mechanism in the liver. As it was expected, the expression of *Per1, Per2,* and *Bmal1* was significantly induced by CR. TR feeding also resulted in an upregulation of the *Per2* at ZT14‐ZT22 and *Bmal1* at ZT22 at the level comparable with the effect of CR but has no effect on the level of *Per1* expression. Thus, periodic fasting contributed to CR‐induced changes in circadian clock gene expression and effects are gene and time of the day dependent.

The circadian clock is a master regulator of metabolism; therefore, changes in circadian rhythms induced by diet might complicate the interpretation of results, especially if the effect of the intervention is assayed at only one time of the day, which, historically, is how it was done for many CR studies. In the current study, mice on all three diets have their major meal approximately at the same time; therefore, we expect that potential variabilities related to the phase of circadian rhythms were minimal. Indeed, both diets, CR and TR, have strong effect on the amplitude and pattern of clock gene expression, but the effect on the phase was small. Thus, the diets significantly affected the circadian rhythms, which might be translated to metabolic changes, but we expect that the induced changes were not trivial consequences of the phase shift.

### 2.4 CR and TR have different effects on blood glucose

**Figure 2:**
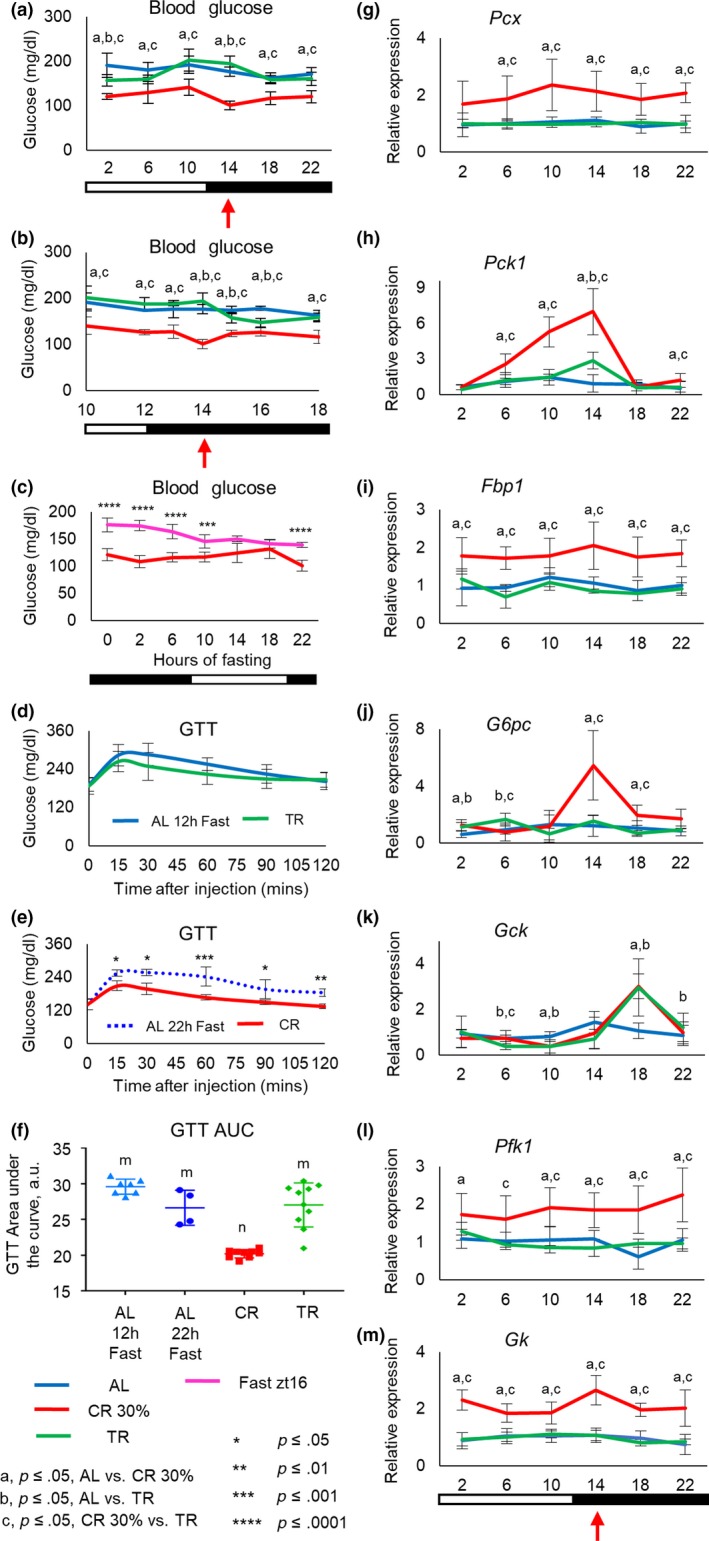
Periodic fasting did not improve glucose homeostasis. (a) Around the clock blood glucose (4‐hr resolution) in AL, CR, and TR mice. The blood glucose was assayed in mice that were not fasted before the experiments. (b) Blood glucose around the feeding time (1‐hr resolution) in AL, CR, and TR mice (*n* = 6–9 per time point per diet). (c) Fasting blood glucose in mice, the food was removed at ZT16, and blood glucose was measured every 4 hr (*n* = 6–8 per time point), (d and e) Intraperitoneal glucose tolerance test (ip‐GTT). Blue solid line—AL mice (*n* = 7) were fasted for 12 hr before the test; blue dotted line—AL mice were fasted for 22 hr before the experiment (*n* = 4); red solid line—CR mice (*n* = 9); green solid line—TR mice (*n* = 10). (f) Area under the curve, the quantification of ip‐GTT data presented in (d and e). The same symbols indicate no statistical significant difference between the diets; different symbols indicate statistical significant difference (*p* < .05) between diets. (g‐m) mRNA expression of rate‐limiting gluconeogenesis genes (g) *Pcx*, (h)* Pck1*, (i)* Fbp1*, and (j) *G6pc;* glycolytic genes (k) *Gck and* (l) *Pfk1;* and glycerol metabolism gene (m) *Gk* in the liver of AL, CR, and TR mice (*n* = 4 per time point per diet). The time of the day when the food was provided for CR and TR mice is indicated by the red arrow. All data represented as Mean ± *SD*. One‐way ANOVA with Bonferroni correction for multiple comparison was performed. Letters and asterix indicate significant effect of the diet (*p* < .05); a—AL versus CR, b—AL versus TR, c—CR versus TR. *p ≤ .05, **p ≤ .01, ***p ≤ .001 and ****p ≤ .0001. Light was turned on at ZT0, and light was turned off at ZT12. Light and dark bars indicate light and dark phases of the day

It is well documented that CR improves glucose homeostasis in rodents (Mitchell et al., 2016). We assayed blood glucose levels around the clock, and the mice were not fasted before the analysis. As expected, CR resulted in reduced blood glucose at all 6 time points across day (Figure 2a and Figure S4). In strong contrast, only a modest reduction in the blood glucose was observed for TR mice at ZT2 and ZT6, but the level was still significantly higher than that for CR. There was no significant difference between AL and TR mice at ZT10‐22. Interestingly, we did not detect any increase in the blood glucose at ZT18, which is just after the feeding for CR and TR mice. Four‐hour resolution in blood glucose measurements might not be sufficient; therefore, blood glucose was assayed with one‐hour resolution for time points ZT12‐ZT16 (Figure 2b and Figure S4). CR group showed some small increase at ZT15‐ZT16 compared with ZT14, and no increase was observed for TR and AL. Thus, the blood glucose level is tightly controlled across the day for all three diets.

Caloric restriction mice were fasted for about 22 hr, and TR mice were fasted for only 12 hr; thus, CR mice were on more severe fasting, which might be the reason for the reduced blood glucose. To assay the effect of acute severe fasting (F) on blood glucose, the food was removed from the group of AL mice that were of the same age as CR mice (5 months). CR mice consumed their meal within two hours; therefore, F was started at ZT16. Blood glucose gradually reduced in F mice with continuation of fasting, and after 18 hr of fasting, it was comparable with levels in CR mice. Contrary to that, CR blood glucose was relatively constant and did not depend on the duration of fasting; in fact, it was slightly increased between 2 and 18 hr of fasting (Figure 2c). Thus, acute severe fasting affected blood glucose, but the effect was different from the effect of CR.

CR mice did not demonstrate any significant change in blood glucose upon feeding (Figure 2b), which suggests a tight regulation. We asked how feeding will affect blood glucose in acute fasted AL mice. AL mice fasted for 22 hr have similar blood glucose with CR mice at ZT14 (145 ± 8.5 mg/dl for fasted AL and 129 ± 25 mg/dl for CR, see also Figure 2c). At ZT14, the same time of the day when 2.4 gram of meal was provided to CR mice, unlimited food was provided to fasted AL mice. Fasted AL mice consumed 0.9 ± 0.01 grams in second hour, which is less than CR mice that consumed all provided 2.4 grams in 2 hr. Blood glucose of refed fasted AL mice was significantly higher than their starting blood glucose or CR blood glucose after feeding. Two hours after feeding (ZT16), the blood glucose was 197 ± 25 mg/dl versus 127 ± 9 mg/dl in CR. Thus, AL mice consumed less carbohydrates, but the blood glucose was higher, which supports the hypothesis that metabolic adaption induced by CR leads to significantly improvement in glucose regulation.

### 2.5 CR but not TR improved glucose tolerance

To further assay the contribution of the diets to glucose homeostasis, we performed intraperitoneal glucose tolerance test (GTT). GTT was performed at ZT14. At this time, CR mice were fasted for about 22 hr and TR mice were fasted for 12 hr; therefore, different controls were used. First, AL mice were fasted for 12 hr, and the food was removed at ZT2, the same time when food was removed for TR mice. Second, AL mice were fasted for 22 hr, and the food was removed at ZT16, the same time when the food was consumed by CR. Starting blood glucose level was different between the groups. Twelve‐hour fasting AL and TR mice have similar starting blood glucose. Twenty‐two‐hour fasting AL and CR mice have similar starting blood glucose, and it was significantly different from starting blood glucose in the other two groups. The kinetics of glucose clearance is presented in Figure 2d‐e, the value at every time point is presented in Figure S6, and area under the curve data for individual mice are presented on Figure 2f. Duration of fasting did not affect glucose clearance response in AL mice (Figure S5). CR mice demonstrated significantly faster blood glucose clearance compared with any AL or TR mouse. In contrast to CR, no difference in GTT was observed between AL and TR mice. Thus, 12‐hr periodic fasting (TR) and acute fasting were not sufficient to improve glucose tolerance. Importantly, our data suggest that fasting blood glucose was not necessary a predictor of improvement in the glucose tolerance and assaying only for fasting blood glucose, as a marker of intervention success, might be misleading.

### 2.6 Glucose metabolism gene expression is regulated by caloric restriction and partially by time‐restricted feeding

Glucose metabolism in the cell is maintained through a chain of reactions that form two biochemical pathways: glycolysis and gluconeogenesis. During the prolonged fasting of CR, several events occur to maintain blood glucose. The energy production switches from carbohydrates to fats; therefore, glycolysis is downregulated and fatty acid oxidation is upregulated under CR. During the first few hours of fasting, glucose is produced predominantly from glycogen and later from other sources, such as amino acids, through gluconeogenesis (Anderson & Weindruch, 2007). The expression of many enzymes involved in glucose metabolism is under circadian control (Eckel‐Mahan & Sassone‐Corsi, 2013; Marcheva et al., 2013), and some of them are also affected by CR (Dhahbi et al., 1999, 2001; Hagopian, Ramsey, & Weindruch, 2008; Hagopian, Soo Hoo, López‐Domínguez, & Ramsey, 2013). Many steps of glycolysis and gluconeogenesis are reversible and catalyzed by the same enzyme, with only a few unique steps to each pathway. To understand the mechanisms of glucose homeostasis under CR and TR diets, we compared the expression of rate‐limiting enzymes across the day in the liver. Glucokinase (*Gck*) and Phosphofructokinase 1 (*Pfk1*) genes encode glycolytic enzymes. mRNA expression for *Gck* was induced at ZT18 for both TR and CR. *Pfk1* mRNA was induced across the day by CR but not by TR (Figure 2k‐L and Table S4). Pyruvate carboxylase (*Pcx*), phosphoenolpyruvate carboxykinase (*Pck1*), fructose‐1,6‐biphosphatase (*Fbp1*), and glucose‐6‐phosphatase (*G6pc*) control irreversible steps in gluconeogenesis. *Pcx* and *Fbp1* expressions were arrhythmic and induced across the day only by CR and not by TR. *Pck1* mRNA expression was rhythmic under all three diets with a peak at ZT10 for AL and at ZT14 for TR and CR. TR resulted in a moderate induction (about 2.5‐fold) at ZT14, and CR caused a strong induction (about fivefold to 10‐fold) at three times: ZT6, ZT10, and ZT14. *G6pc* expression was arrhythmic under AL and TR, and it became rhythmic under CR with a peak at ZT14 (Figure 2g–j and Table S4). We also measured mRNA expression of glycerol kinase (*Gk*), which plays an important role in glycerol metabolism, and a previous study demonstrated how CR affects glycerol metabolism (Hagopian et al., 2008); *Gk* was also induced across the day upon CR and not by TR (Figure 2m and Table S4). Thus, periodic fasting contributed to CR‐induced changes in *Gck* expression and, to some extent, *Pck1* expression was regulated by both periodic fasting and by caloric intake. We cannot conclude at this point whether *Fbp1, Pfk1*, *Pcx*, *G6pc, and Gk* were regulated exclusively by reduced caloric intake or fasting because the duration of fasting was different between CR and TR. Additional studies are necessary. CR‐induced changes in mRNA expression in our study are in agreement with previously reported induction of the same enzymes at both transcription and enzyme activity levels (Dhahbi et al., 2001); therefore, we might expect that increased mRNA expression will result in increased expression on protein level and can contribute to the improved glucose homeostasis under CR.

### 2.7 Effect of diets on blood insulin and on insulin tolerance

**Figure 3:**
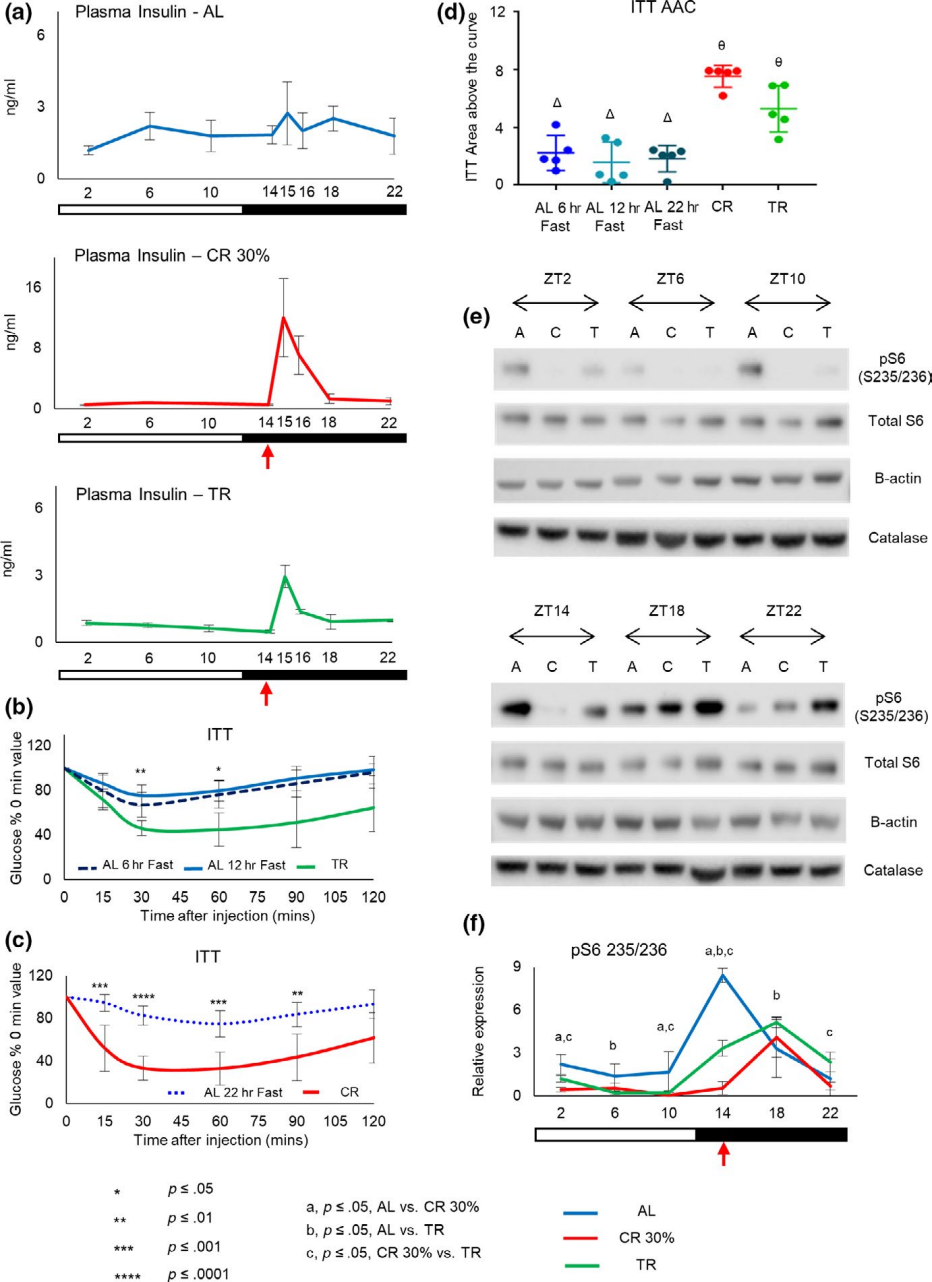
Periodic fasting contributes to CR induced improved insulin homeostasis and downregulation of mTORC1. (a) Around the clock plasma insulin levels. AL (upper panel), CR (middle panel), and TR (lower panel) mice (*n* = 4 per time point per group). The insulin was measured in blood obtained from tail vein. Mice on all three diets were not fasted prior to the experiment. (b‐c) Intraperitoneal Insulin tolerance test (ip‐ITT). Blue dashed line—AL mice fasted for 6 hr (*n* = 5); blue solid line—AL mice fasted for 12 hr (*n* = 5); blue dotted line—AL mice fasted for 22 hr (*n* = 4); green solid line—TR mice (*n* = 5); red solid line—CR mice (*n* = 8). (d) Area above the curve, the quantification of ip‐ITT data presented in (b and c). The same symbols indicate no statistical significant difference between the diets; different symbols indicate statistical significant difference (*p* < .05) between diets. (e) Circadian rhythms of ribosomal protein S6 phosphorylated on serine 235/236 in the liver of mice on different diets; (e) representative Western blot and (f) quantification (*n* = 4 per time point per diet). The time of the day when the food was provided for CR and TR mice is indicated by the red arrow. All data represented as Mean ± *SD*. One‐way ANOVA with Bonferroni correction for multiple comparison was performed. Letters and asterix indicate significant effect of the diet (*p* < .05); a—AL versus CR, b—AL versus TR, c—CR versus TR. *p ≤ .05, **p ≤ .01, ***p ≤ .001 and ****p ≤ .0001. Light was turned on at ZT0, and light was turned off at ZT12. Light and dark bars indicate light and dark phases of the day

Insulin is one of the key hormones in glucose homeostasis. We measured plasma insulin levels across the day in all 3 diets (Figure 3a and Figure S7). Daily rhythms in plasma insulin are shown in Figure 3a. For AL mice (upper panel), the insulin oscillated with low amplitude and it was slightly higher during the dark phase of the day. For CR mice (middle panel) and TR mice (lower panel), the insulin oscillated with high amplitude and according to the feeding patterns of mice on these diets. As it was expected, insulin was low during the light phase (ZT2‐ZT14) and it was significantly induced after the feeding (ZT15‐ZT16), and after that (ZT18‐ZT22), the insulin dropped down. Comparison of the diets at every time point is presented in Figure S7. During light phase of the day (ZT2‐14), there was no difference in plasma insulin between CR and TR groups and both were significantly lower than AL mice. This reduction was expected because it is the period of fasting for TR and CR mice, and it agrees with previously reported reduced insulin levels under CR (Masoro et al., 1992). After feeding, the insulin increased in both CR and TR but with different kinetics and magnitudes. There was about a 25‐fold increase for CR group at ZT15 and a 15‐fold increase at ZT16. Thus, during this time, blood insulin in CR mice exceeded the AL insulin level. The sharp induction in plasma insulin agrees with the phase of active feeding. For TR mice, the induction was not as strong (about an eightfold increase at zt15 and a threefold increase at zt16) compared to CR; however, the induction of insulin upon CR and TR correlates with their feeding pattern. Thus, periodic fasting strongly contributed to the effect of CR on insulin levels, and the effect is time of the day dependent.

Caloric restriction significantly improves insulin sensitivity (Masoro et al., 1992; Mitchell et al., 2016). Intraperitoneal insulin tolerance test (ITT) was performed at ZT14. AL mice fasted for 6 hr were controls for TR, and 22‐hr fasted AL mice were controls for CR mice. Absolute values of blood glucose are presented in Figure S9, values normalized to starting blood glucose at time 0 are presented in Figure 3b and c, and values for individual animals at every time point are in Figure S8. Area above the curve (AAC) are shown for individual mice in Figure 3d. CR mice demonstrated the greatest insulin sensitivity, and TR mice have sensitivity lower than CR but higher than AL mice fasted for 6 hr, 12 hr, or 22 hr, while no difference between three groups of fasted AL mice was detected. The reduced insulin level observed in TR and CR mice agrees with the increased insulin sensitivity under these diets.

Glucose homeostasis and insulin are tightly interlinked and central to the mechanisms of CR (Masoro et al., 1992; Mitchell et al., 2016). TR reduced blood insulin levels and improved insulin sensitivity similar with CR. At the same time, TR did not significantly reduce blood glucose and did not improve glucose tolerance contrasting with CR. Blood glucose levels are regulated by glucose uptake from the diet, glucose production through gluconeogenesis, and glucose tissue uptake. Insulin is involved in the regulation of glucose production and uptake; therefore, the differential effect on blood glucose and insulin might look surprising. We suspect that improvement in insulin sensitivity contributes to the improvement in glucose homeostasis under CR, but several factors might also be important. One factor is the difference in the effect of the diets on insulin production/secretion. GTT experiment started at ZT14 and continued until ZT16. CR, but not TR, mice have significant increase in blood insulin at this time; therefore, one might expect a higher level of insulin secretion in response to glucose injection in CR mice during this time and, as a result, a better ITT performance for CR mice even if the insulin sensitivity is improved for both CR and TR.

### 2.8 Effect of diets on mTORC1 signaling pathway

Mechanistic target of rapamycin (mTOR) signaling pathway plays an important role in aging and in the mechanism of CR. CR reduces the activity of mTOR complex 1 (mTORC1) in different organisms (Kapahi et al., 2010; Powers, Kaeberlein, Caldwell, Kennedy, & Fields, 2006). In mammals, the effect of CR is time of the day dependent (Tulsian et al., 2018). Insulin is a major regulator of mTORC1 activity in the liver. We observed a significant effect of diets on plasma insulin; therefore, we decided to compare the effect of the diets on activity of mTORC1. The phosphorylation of ribosomal protein S6 was assayed as a surrogate marker of mTORC1 activity (Figure 3e and f). For all three diets, the phosphorylation of S6 was rhythmic with high levels during the dark phase and low levels during the light phases; the peak for AL was at ZT14 and for both TR and CR at ZT18; and thus, diets have a minimal effect on the phase of S6 phosphorylation. During the light phase, AL mice had access to food and were eating while both TR and CR mice were fasting. During this time, mTORC1 activity was significantly higher in the liver of AL mice compared with CR or TR. ZT18 coincided with the end of the first large meal for AL and TR mice, whereas CR mice consumed all their daily intake by this time (see Figure 1f). mTORC1 activity was high for all three diets at ZT18 in agreement with active feeding. CR mice have high mTORC1 activity only at ZT18 while AL and TR mice have broader peaks with high activity between ZT14 and ZT22, again, in good agreement with feeding pattern and rhythms in plasma insulin. Interestingly, at ZT14 mTORC1 activity was high in the liver of AL and TR but not CR mice. High activity for AL group was not surprising because these mice started active feeding around ZT12‐ZT13. Tissues of TR mice at ZT14 were collected before the feeding; therefore, the high level of mTORC1 activity might represent food anticipation by the liver. However, tissue for CR mice was also collected before feeding indicating that mTORC1 activity is affected differently by CR. Thus, periodic fasting contributes to downregulation of mTORC1 activity by CR during the light phase of the day.

In conclusion, our study highlights that some, but not all, of the metabolic benefits observed in caloric restriction can be achieved by periodic fasting. This observation is in agreement with a recent study by Mitchell et al., 2019. CR increases mouse lifespan by 28%, and mealtime feeding increases lifespan by only 11%–14%. For unknown reasons, mealtime fed mice eat their daily amount of food during the restricted time window; thus, mealtime is also a form of self‐implemented TR. Interestingly, the fasting period in both Mitchell et al., 2019 and our study is very similar around 12 hr. The increase in lifespan is achieved without reduction in food intake or body weight which correlates with increased insulin sensitivity in our TR mice. Increased insulin sensitivity was proposed as an important contributing factor to longevity under CR; thus, our study may provide some mechanistic explanation to the increase in longevity induced by mealtime feeding.

The current study has several limitations that need to be addressed in the future. The food intake was reduced by 30%; thus, all nutrients were reduced by 30%. This brings us to an important question: whether the reduction in blood glucose can be explained through 30% reduction in carbohydrate intake. Effects of diet composition on metabolism and longevity, so‐called nutritional geometry, are growing field (Simpson et al., 2017). However, the development of strategy to test the hypothesis on carbohydrate reduction is challenging. There are multiple ways to change carbohydrate density of the diet, and a connection between carbohydrate density of the food and blood glucose is complicated (Garbow et al., 2011; Grandl et al., 2018; Kinzig, Honors, & Hargrave, 2010; Meidenbauer, Ta, & Seyfried, 2014). A separate controlled study is necessary to understand the relative contribution of reduction in individual nutrients on glucose homeostasis. The study was focused on glucose homeostasis, and we did not compare the effect of diets on several signaling pathways such as sirtuin, insulin signaling, or IGF‐1 signaling pathways which are known to play an important evolutionary conserved role in the mechanisms of aging and CR along with their linkage to the circadian clock. The study was focused on the liver while skeletal muscle and adipose tissue are also important contributors to glucose homeostasis. GTT and ITT were performed only at one time of the day, before the feeding, and the response might be different at a different time, for example, after the feeding. Most of the experiments were performed with male mice, and some of the effects might be different in females. Finally, the effect of severe periodic fasting and the effect of discrete meals for CR need to be studied in the future to better understand the contribution of periodic fasting to mechanisms of CR.

## 3 EXPERIMENTAL PROCEDURES

### 3.1 Experimental model and subject details

#### 3.1.1 Animals

All experiments involving animals were conducted in accordance with federal and university guidelines, and all procedures were approved by IACUC, Cleveland State University. C57BL/6J mice were bred in‐house at Cleveland State University. Mice were maintained on 12‐hr light: 12‐hr dark cycle (LD12:12) with lights on at 7 a.m. (ZT0) and lights off at 7 p.m. (ZT12). Animals were maintained in groups of four animals per cage throughout the experiment. All mice were fed 2018 Teklad global 18% protein rodent diet (Envigo (formerly Harlan), Cat# 2018, Madison, WI). Mice used in the experiments were 16 weeks of age at the start of the experiments.

### 3.2 Method details

#### 3.2.1 Study design

Prior to the start of the experiment, all mice were fed ad libitum amount of food. At 16 weeks of age, mice were randomly assigned to three groups. One group of mice continued ad libitum diet (AL), and second group of mice were subjected to 30% caloric restriction (CR). In this group, restriction in the amount of provided food was introduced gradually; 10% reduction was implemented during the first week, followed by 20% reduction in the second week, and finally 30% reduction until the end of experiment. CR group received food once per day at ZT14 (9 p.m.). Third group of mice was on time‐restricted feeding (TR). Food was provided to TR mice at ZT14 (9 p.m.), and food was withdrawn at ZT2 (9 a.m.) every day; thus, TR mice were fed ad libitum amount of food for 12 hr per day. Mice on all three diets had unlimited access to water. Body weight and food intake for mice on CR and TR diets were measured once per day at the same time of day for the first ten days and after that once per week until the end of the experiment. Body weight measurements for mice on AL diet were made as mentioned above; food intake for the first ten days was not measured in AL group; however, weekly measurements were made until the end of the experiment. Tissue harvesting was performed after 8–10 weeks on the diets, and all collected tissues were frozen immediately on dry ice and stored at −80°C until the analysis. Tissues were collected around the clock with four‐hour resolution at ZT2, 6, 10, 14, 18, and 22.

### 3.3 Daily food intake pattern

Mice were acclimatized for 5 weeks to the diets. Measurements of food intake were performed simultaneously in all three groups. Mice were provided with preweighed amount of food; food was removed, weighed, and placed back every hour for 24 hr. The reduction in the weight of the food was taken as amount of consumed food. This amount was divided on the number of animals in the cage to calculate the amount of hourly food intake per mouse.

### 3.4 Blood glucose measurement

Blood for the analysis was collected through the tail vein nick at time points ZT2, 6, 10, 14, 18, and 22 and for kinetics experiment from ZT12‐18 with one‐hour resolution. Blood glucose was measured using CVS Advanced Health Blood Glucose Meter (CVS Pharmacy, Woonsocket, RI) with CVS Health Advanced Glucose Meter Test Strips (CVS Pharmacy, Woonsocket, RI).

### 3.5 Intraperitoneal glucose and insulin tolerance tests (GTT and ITT)

After 5–8 weeks on the diets, mice were subjected to glucose tolerance and insulin tolerance tests. For GTT experiment, mice were fasted for 12 hr and intraperitoneally injected with glucose (0.4 g/kg body weight, Fisher, Hampton, NH). For ITT experiment, mice were fasted for 6 hr and intraperitoneally injected with insulin (0.06 U/kg body weight, Humulin® R U‐100, Indianapolis, IN), respectively. Blood glucose was assayed as above before and after injection through regular intervals as indicated. Both ip‐GTT and ip‐ITT experiments were performed at ZT14.

### 3.6 Plasma insulin assay

Blood was collected through the tail vein nick and immediately mixed with EDTA to prevent coagulation, samples were centrifuged at  4,200 *g* for 20 min at 4°C, and supernatant was used for the analysis. Blood plasma insulin was measured using commercially available mouse ultra‐sensitive ELISA kit (Crystal Chem, Downers Grove, IL).

### 3.7 RNA isolation and processing

Total RNA was extracted from frozen liver tissue using TRIzol according to manufacturer's instructions. Briefly, frozen tissue was mixed with 1ml of TRIzol reagent (Invitrogen™, Carlsbad, CA), homogenized using sonicator, and centrifuged at 15,300 *g* for 10 min. Supernatant from this solution was transferred to new tube and mixed with 200 µl chloroform, shaken, and centrifuged at 14,000 *g* for 15 min at 4°C. The aqueous phase was separated and mixed with equal amount of isopropanol, shaken, and centrifuged at 20,800 *g* for 10 min at 4°C. The RNA pellet was washed with 1ml of 75% ethanol prior to re‐suspending the pellet in 30µl RNase‐free water. Quantification of RNA was performed using Nanodrop‐2000 (Thermo Fisher Scientific Inc., Waltham, MA) and quality of total RNA checked by gel electrophoresis.

### 3.8 Analysis of mRNA expression with real‐time quantitative PCR

After quantification and quality check of total RNA by electrophoresis, 1,000 ng of total RNA was reverse transcribed using SuperScript IV Reverse Transcriptase (Invitrogen™, Carlsbad, CA). Real‐time quantitative PCR was performed using iTaq Universal SYBR Green Supermix (Bio‐Rad, Hercules, CA) on CFX Connect real‐time PCR detection instrument (Bio‐Rad, Hercules, CA). For list and details of primers, refer to Table S1. 18S rRNA expression levels were used for normalization. Fold change was determined by ΔΔCt method.

### 3.9 Analysis of protein expression with western blotting

Total liver lysates were prepared with Cell Signaling Buffer (1M Tris base pH 7.5, 5M NaCl, 0.5M EGTA, 0.5M EDTA, Triton‐X, 0.1M Na_4_P_2_O_7_, 1M β‐glycerophosphate, 1M Na_3_VO_4_) containing protease and phosphatase inhibitor cocktails (Sigma‐Aldrich, St. Louis, MO). The homogenates complete with SDS loading mix were loaded in 4%–12% Bis‐Tris NUPAGE gels (Thermo Fisher Scientific Inc., Waltham, MA). After electrophoretic run, proteins were transferred onto PVDF membrane (Thermo Fisher Scientific Inc., Waltham, MA) and blocked in 5% milk prepared in TBS with 0.1% Tween‐20 for 1 hr. Incubation with primary antibodies was done overnight with gentle shaking at 4°C. For list of antibodies, please refer to Table S2. β‐Actin (Sigma‐Aldrich, St. Louis, MO) was used as internal control. Catalase (Santa Cruz Biotechnology, Dallas, TX) was used as additional loading control. Quantification of images was done using Image Studio Lite software (LI‐COR Biosciences, Lincoln, NE).

### 3.10 Quantification and statistical analysis

Data for all three diets were analyzed using ordinary one‐way ANOVA (multiple comparison correction was done using Bonferroni method). All statistical analysis was performed using GraphPad Prism 7.0. Exact number of mice per diet group for every experiment is reported in Figure legend. Data are shown as Mean ± *SD*. *p* ≤ .05 was considered as statistically significant difference between the diets. The circadian rhythmicity of genes expression was determined by JTK‐Cycle (Hughes, Hogenesch, & Kornacker, 2010). The cutoff for adj. p value was set at ≤0.05 for circadian rhythmicity.

## CONFLICT OF INTEREST

The authors declare no competing interests.

## AUTHOR CONTRIBUTIONS

N.V. and R.K. conceived and designed the study. All authors contributed to the performing experiments. N.V. and R.K. contributed to analysis and interpretation of data. N.V. and R.K. wrote the manuscript. All authors contributed to reading, revision, and approval of manuscript.

## Supporting information

Fig S1‐S9

Table S1‐S4

## Data Availability

The data that support the findings of this study are openly available in Mendeley Data at http://dx.doi.org/10.17632/8nczbhd7xc.2.
