# Supplementary material for: Reduced caloric intake and periodic fasting independently contribute to metabolic effects of caloric restriction
Source: Aging Cell. 2020 Mar 11;19(4):e13138. doi: 10.1111/acel.13138 (PMC7189989; doi:10.1111/acel.13138)
Supplement: Supplementary file 1 — Fig S1‐S9 [file ACEL-19-e13138-s001.docx]

**Supplemental Figures**

**Supplemental Figure 1**


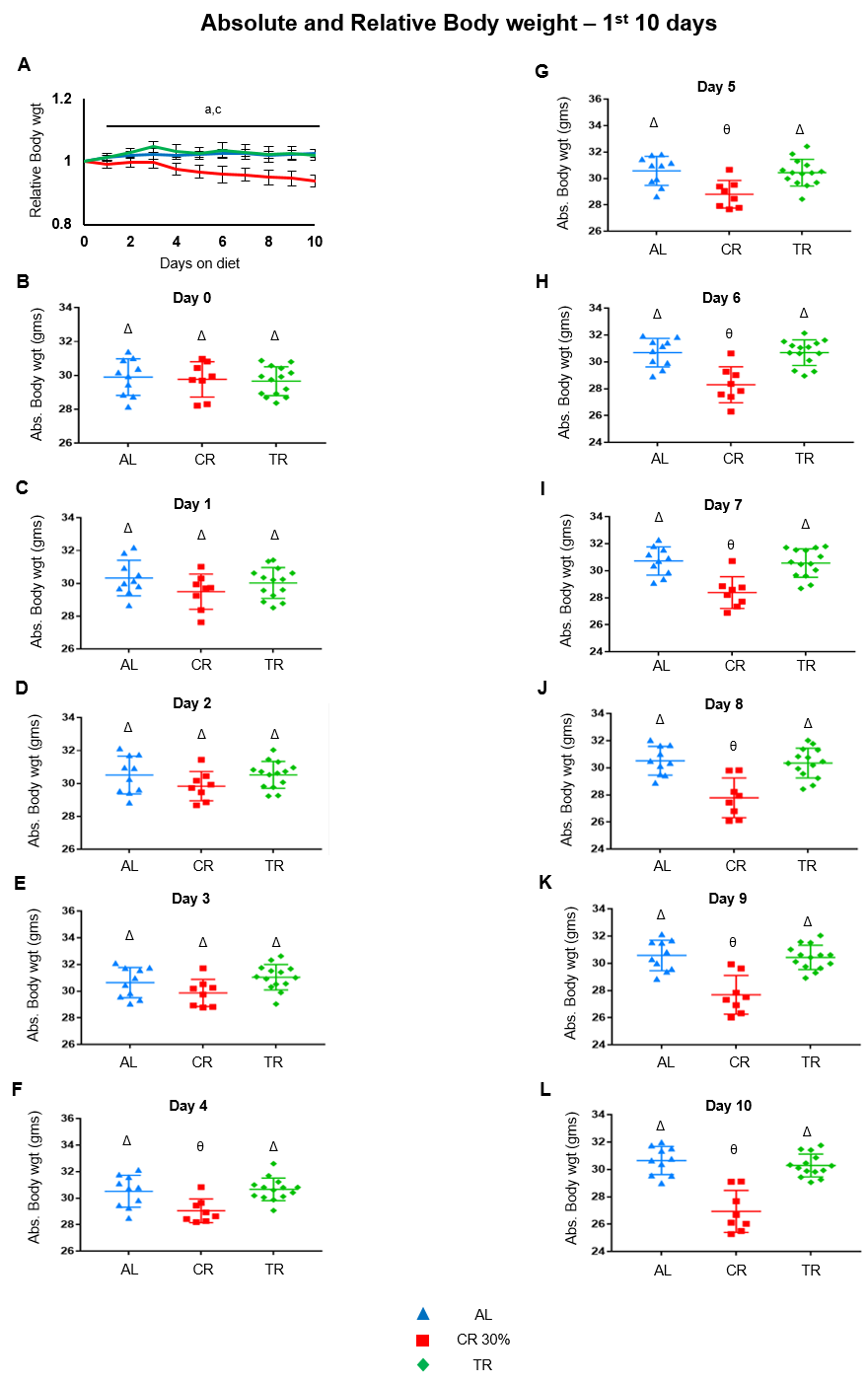


**Figure S1** – **related to Figure 1: Effect of diets on body weight for first ten days from the start of the experiment.**

(A) Relative body weight and (B-L) Absolute body weight of mice on AL (n=10), CR (n=8) and TR (n=14), represented in the form of scatter plot, during the first ten days from the start of the experiment. (A): AL - blue solid line, blue solid triangles; CR – CR - red solid line, red solid squares; TR – green solid line, green solid diamonds. Diet groups with the same letters indicate no statistical significant effect of diets. Diet groups with different letters indicate statistical significant effect of diets. p<0.05 considered as statistical significance.

**Supplemental Figure 2**


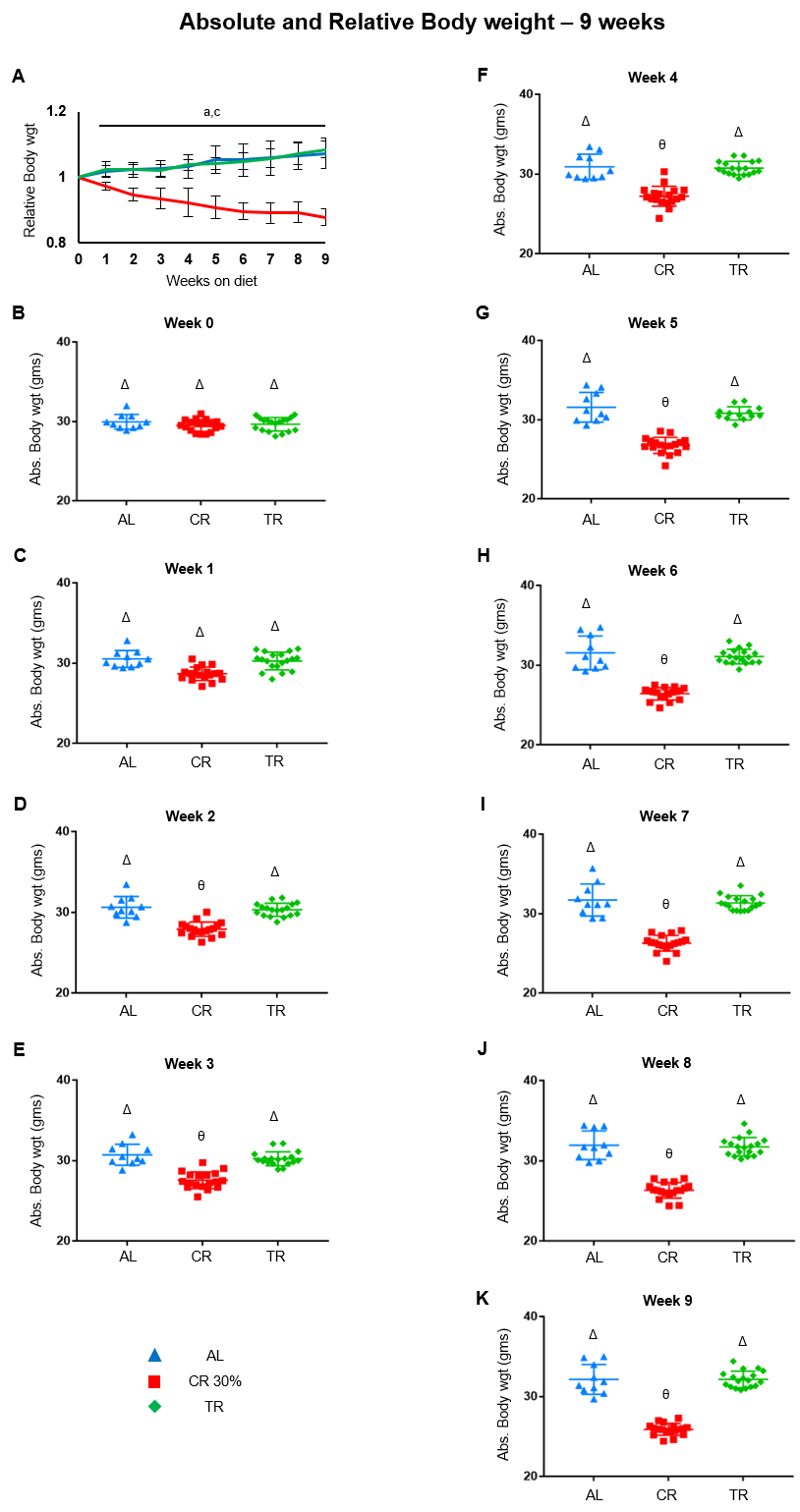


**Figure S2** – **related to Figure 1: Body weight remains unchanged under TR.**

(A) Relative body weight and (B-K) Absolute body weight of mice on AL (n=10), CR (n=18) and TR (n=18) measured once a week until the end of the experiment. AL – blue solid line, blue solid triangles; CR – red solid line, red solid squares; TR – green solid line, green solid diamonds. Diet groups with the same letters indicate no statistical significant effect of diets. Diet groups with different letters indicate statistical significant effect of diets. p<0.05 considered as statistical significance.

**Supplemental Figure 3**


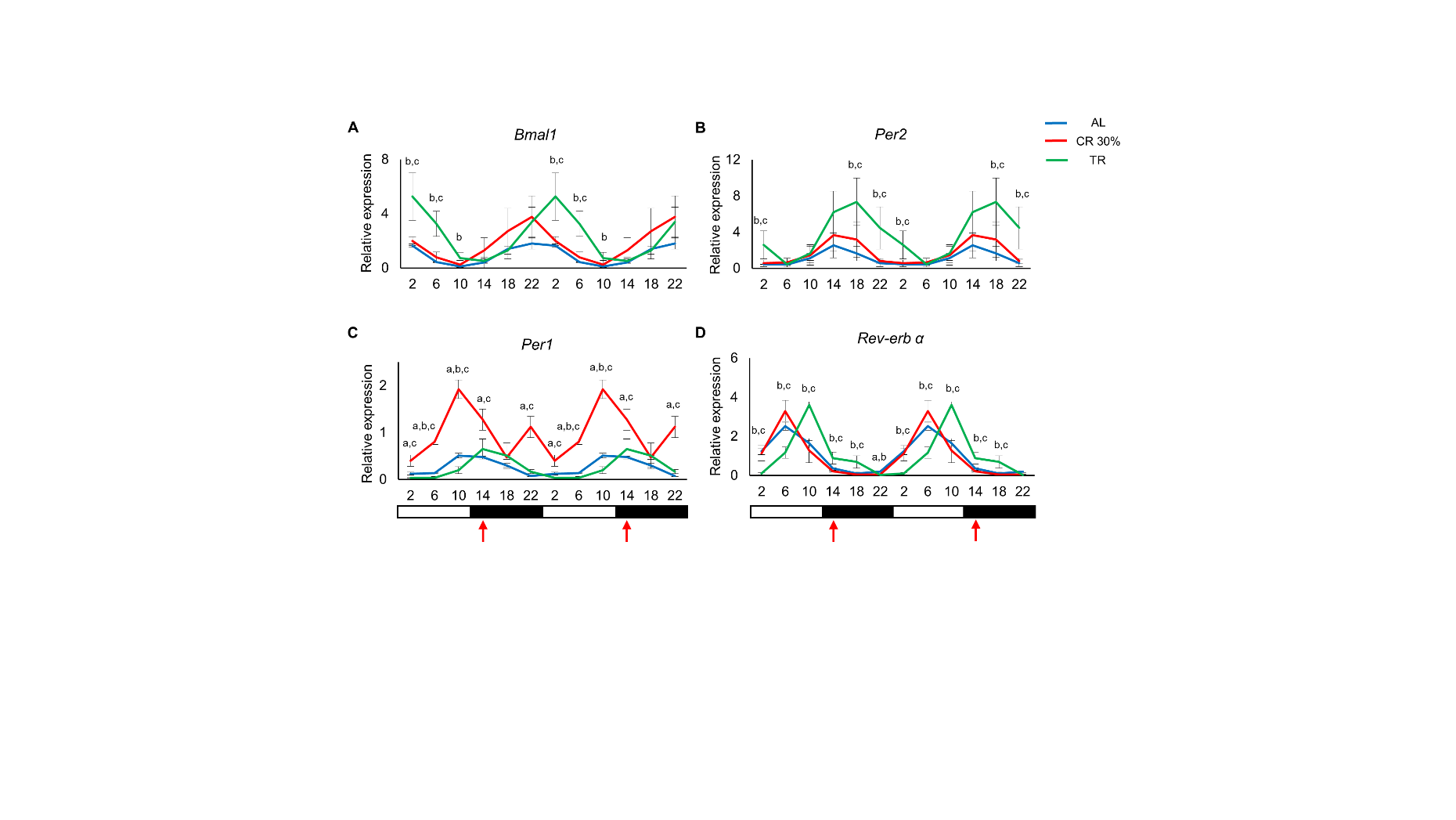


**Figure S3 – related to Figure 1: CR and TR do not disturb clock mechanism in the liver.**

mRNA expression of circadian clock genes: *Bmal1* (A), *Per2* (B), *Per1* (C) and *Rev-erb α* (D) in the liver. AL - blue solid line; TR -green solid line; CR - red solid line (n=4 per time point per diet). Data for clock genes was double plotted for illustrative purposes.

The time of the day when the food was provided for CR and TR mice is indicated by red arrows. Letters indicate significant effect of the diet (p<0.05); a – AL versus CR, b – AL versus TR, c – CR versus TR. Light was turned on at ZT0 and light was turned off at ZT12. Light and Dark bars indicate light and dark phases of the day.

**Supplemental Figure 4**


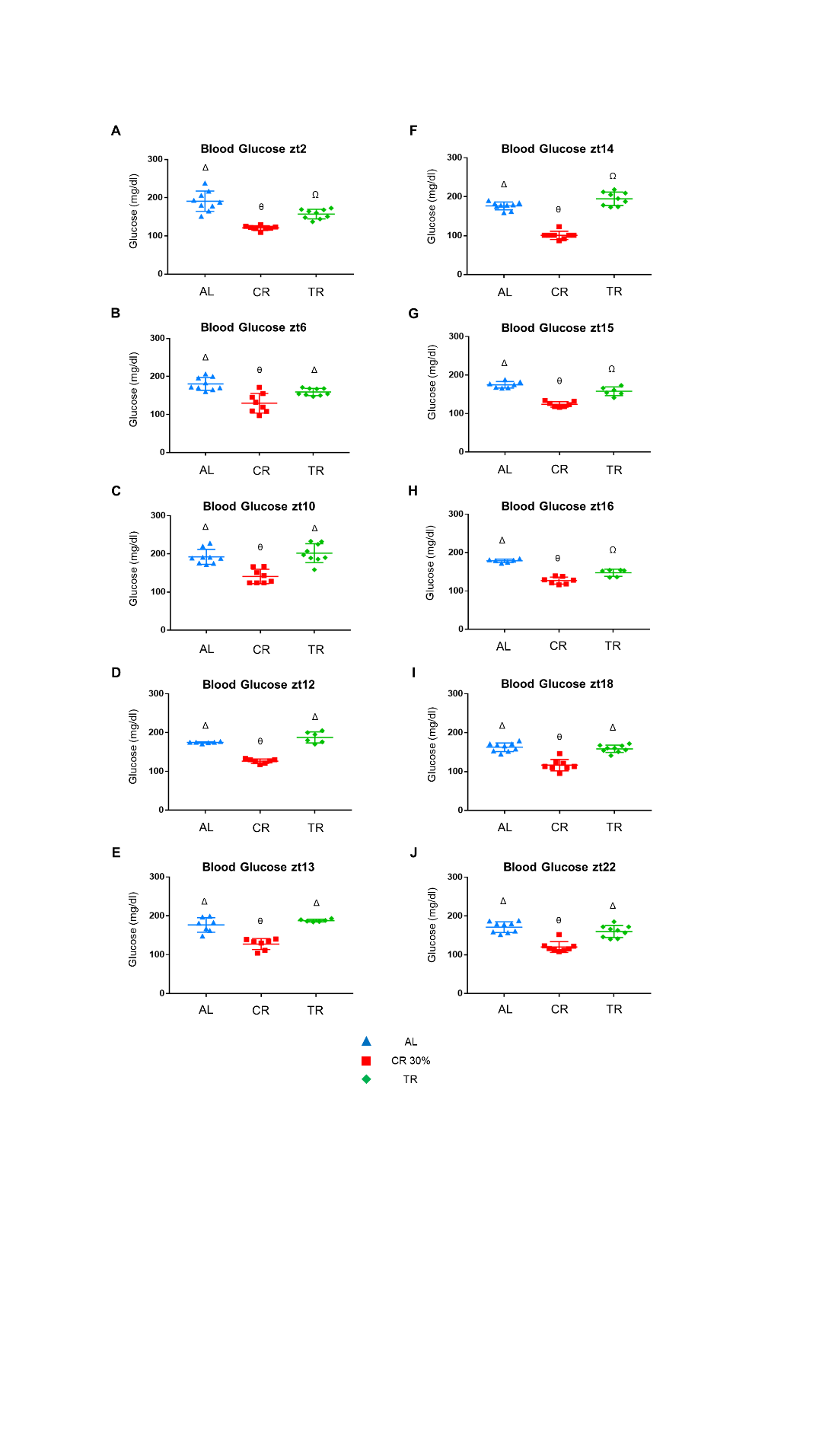


**Figure S4** – **related to Figure 2: Effect of diet on blood glucose in mice.**

(A-J) Scatter plot representation of blood glucose at individual time points obtained from tail vein of mice on AL, CR and TR. For time points zt2,6,10,14,18,22, AL (n=9), CR (n=8) and TR (n=9); and for time points zt12,13,15 and 16, AL (n=6-7), CR (n=7) and TR (n=6). AL – blue solid triangles; CR – red solid squares; TR – green solid diamonds. Diet groups with same letters indicate no statistical significant effect of diets. Diet groups with different letters indicate statistical significant effect of diets. p<0.05 considered as statistical significance.

**Supplemental Figure 5**

**
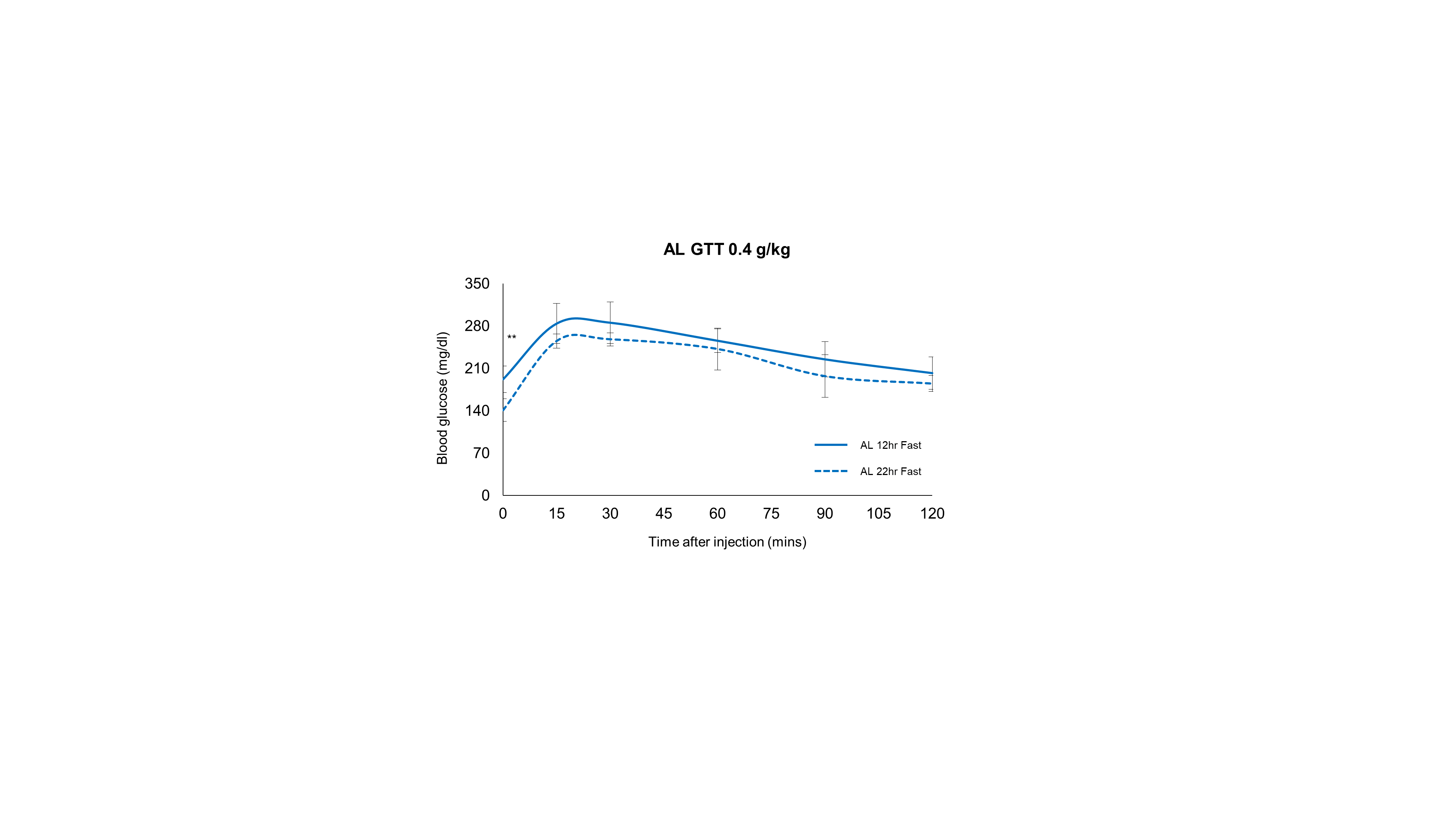
**

**Figure S5 – related to Figure 2: Duration of fasting does not affect GTT.**

Blood glucose values obtained from tail vein of mice subjected to different durations of fasting (12hr and 22hr) under AL feeding regimen for glucose tolerance test. AL 12hr Fast (n=7) – blue solid line; AL 22hr Fast (n=4) – blue dashed line. Asterisks indicate statistical significant effect of the diets: * - P ≤ 0.05, ** - P ≤ 0.01, *** - P ≤ 0.001 and **** - P ≤ 0.0001.

**Supplemental Figure 6**


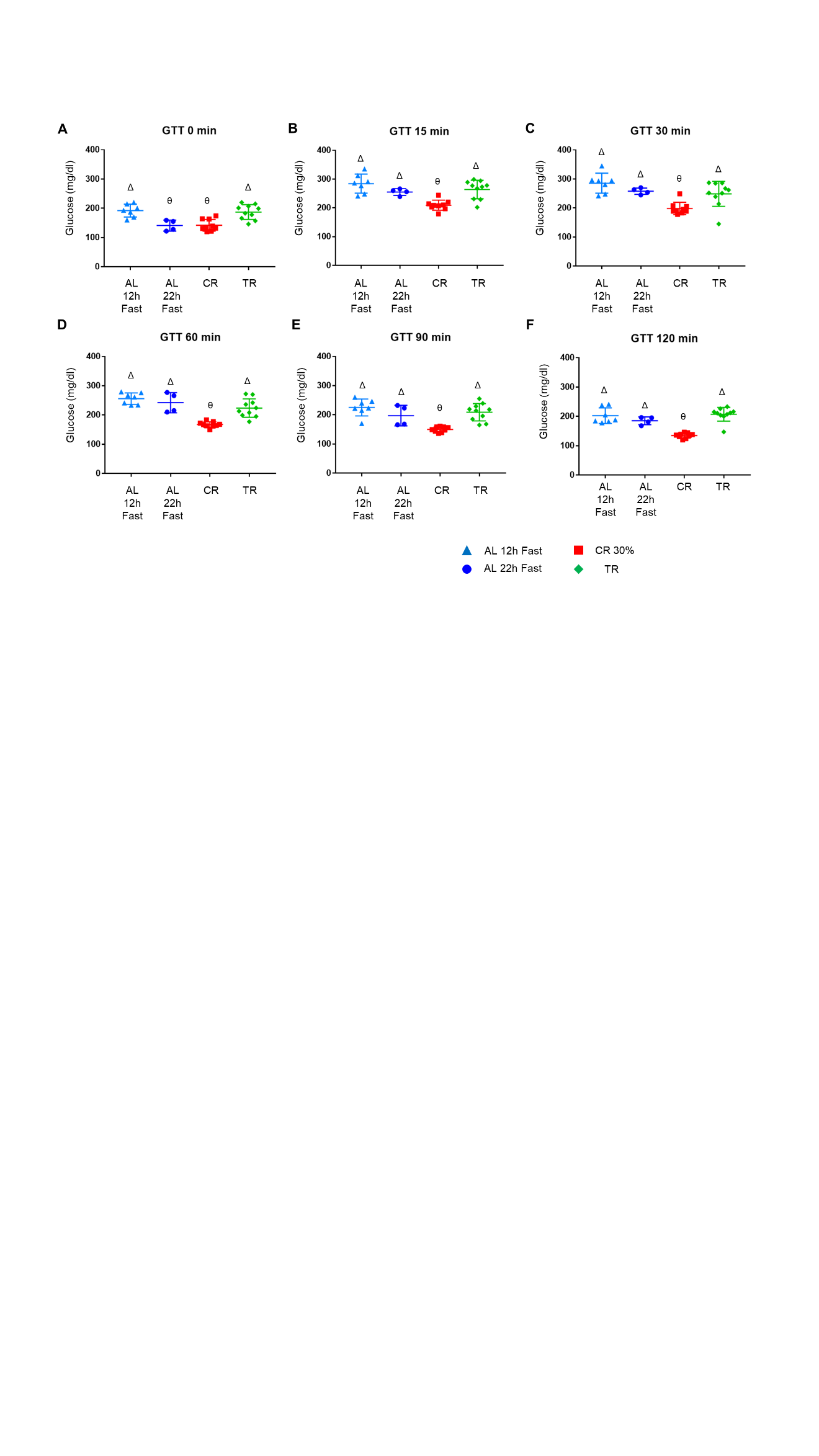


**Figure S6** – **related to Figure 2: TR does not affect GTT.**

(A-J) Scatter plot representation of blood glucose levels at individual time points obtained from tail vein of mice on AL 12hr Fast (n=7), AL 22hr Fast (n=4), CR (n=9) and TR (n=10) during glucose tolerance test (ip-GTT). AL 12hr Fast – blue solid triangles; AL 22hr Fast – blue solid circles; CR – red solid squares; TR – green solid diamonds. Diet groups with the same letters indicate no statistical significant effect of diets. Diet groups with different letters indicate statistical significant effect of diets. p<0.05 considered as statistical significance.

**Supplemental Figure 7**


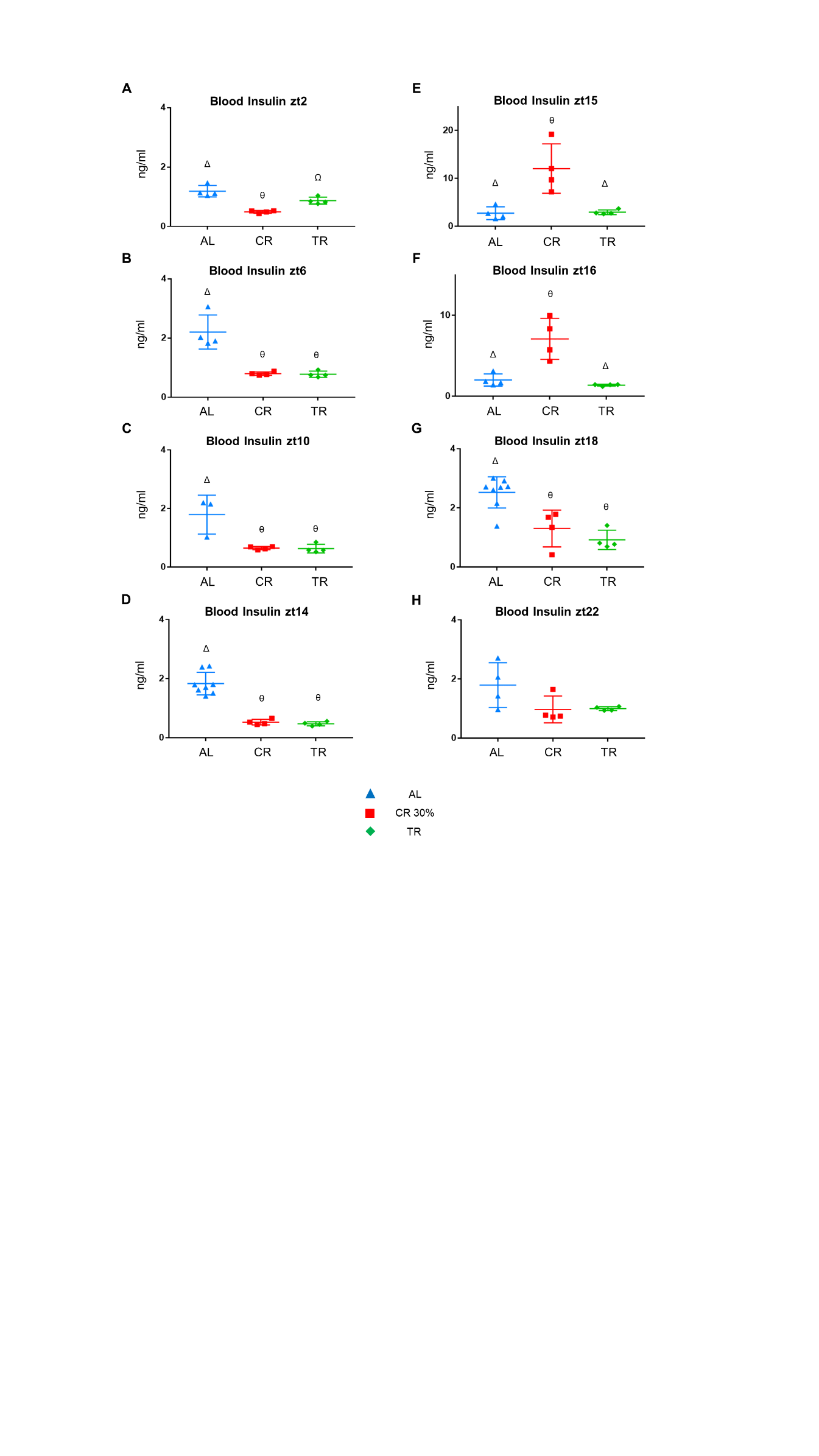


**Figure S7** – **related to Figure 3: TR and CR regulate blood insulin levels.**

(A-H) Scatter plot representation of blood insulin levels at individual time points obtained from tail vein of mice on AL (n=4, except zt14 and zt18 (n=8)), CR (n=4) and TR (n=4). AL – blue solid triangles; CR – red solid squares; TR – green solid diamonds. Diet groups with the same letters indicate no statistical significant effect of diets. Diet groups with different letters indicate statistical significant effect of diets. p<0.05 considered as statistical significance.

**Supplemental Figure 8**


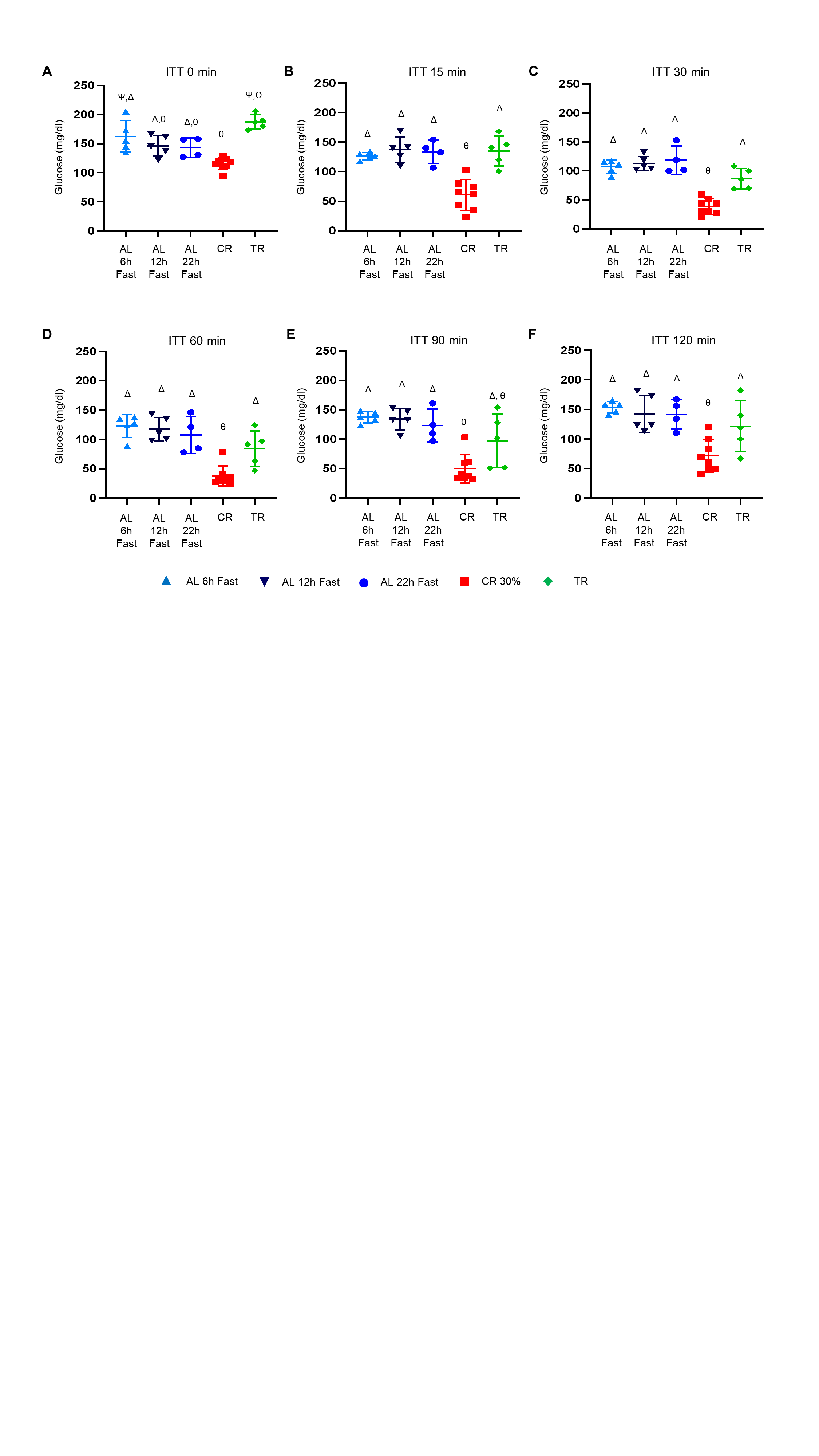


**Figure S8** – **related to Figure 3: CR and TR contribute to improvement in insulin sensitivity.**

(A-J) Scatter plot representation of blood glucose levels obtained from tail vein of mice on AL 6hr Fast (n=5), AL 12hr Fast (n=5), AL 22hr Fast (n=4), CR (n=8) and TR (n=5) during insulin tolerance test (ip-ITT) at individual time points. AL 6hr Fast – blue solid triangles; AL 12hr Fast – blue solid inverted triangles; AL 22hr Fast – blue solid circles; CR – red solid squares; TR – green solid diamonds. Diet groups with the same letters indicate no statistical significant effect of diets. Diet groups with different letters indicate statistical significant effect of diets. p<0.05 considered as statistical significance.

**Supplemental Figure 9**


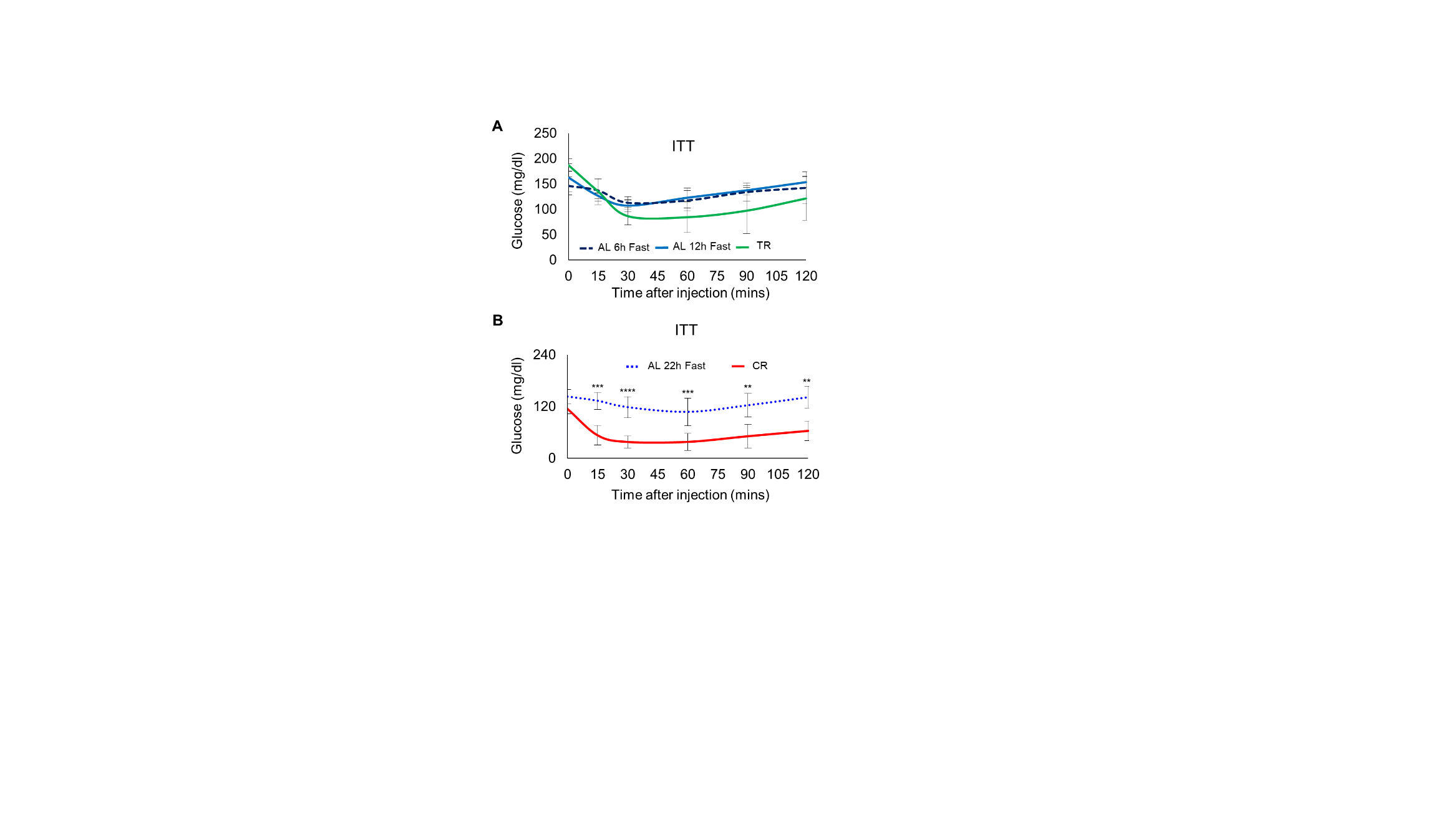


**Figure S9 – related to Figure 3: Insulin sensitivity is improved upon CR and TR diets.**

(A-B) Absolute blood glucose values during insulin tolerance test obtained from tail vein of mice subjected to AL 6hr Fast, AL 12hr Fast, AL 22hr Fast, CR and TR diets. AL 6hr Fast (n=5) – blue dashed line; AL 12hr Fast (n=5) – blue solid line; AL 22hr Fast (n=4) – blue dashed line; CR (n=8) – red solid line; TR (n=5) – green solid line. Asterisks indicate statistical significant effect of the diets: * - P ≤ 0.05, ** - P ≤ 0.01, *** - P ≤ 0.001 and **** - P ≤ 0.0001.
