# Supplementary material for: Reduced caloric intake and periodic fasting independently contribute to metabolic effects of caloric restriction
Source: Aging Cell. 2020 Mar 11;19(4):e13138. doi: 10.1111/acel.13138 (PMC7189989; doi:10.1111/acel.13138)
Supplement: Supplementary file 2 — Table S1‐S4 [file ACEL-19-e13138-s002.docx]

**Supplementary Tables**

**Supplemental Table 1: List of primer sequences used for Real Time quantitative PCR**

| **Gene** | **Company** | **Forward and Reverse Primer Sequences** |
| --- | --- | --- |
| *Bmal1* | IDT | Fwd 5’ CACTGTCCCAGGCATTCCA 3’  Rev 5’ TTCCTCCGCGATCATTCG 3’ |
| *Per1* | IDT | Fwd 5’ AGGTGGCTTTCGTGTTGG 3’  Rev 5’ CAATCGATGGATCTGCTCTGAG 3’ |
| *Per2* | IDT | Fwd 5’ AGGCACCTCCAACATGCAA 3’  Rev 5’ GGATGCCCCGCTTCTAGAC 3’ |
| *Rev-erbα* | IDT | Fwd 5’ TGGCCTCAGGCTTCCACTATG 3’  Rev 5’ CCGTTGCTTCTCTCTCTTGGG 3’ |
| *Fmo3* | IDT | Fwd 5’ CACCACCATCCAGACAGATTAC 3’  Rev 5’ CCTTGAGAAACAGCCATAGGAG 3’ |
| *Serpina12* | IDT | Fwd 5’ ACCGTGATGATTCTCACAAA 3’  Rev 5’ AACATCATGGGTACCTTCAC 3’ |
| *Mup4* | IDT | Fwd 5’ ACCAAAACCAATCGCTGCCT 3’  Rev 5’ GCTGTATCGATCGGAAGAGAGG 3’ |
| *Cyp4a12b* | IDT | Fwd 5’ CTGATGGACGTTCTTTAC 3’  Rev 5’ TCAAACACCTCTGGATT 3’ |
| *Gck* | IDT | Fwd 5’ CACAATGATCTCCTGCTACT 3’  Rev 5’ TTCTGCATCTCCTCCATGTA 3’ |
| *Pfk1* | IDT | Fwd 5’ AGAGGACCTTTGTTTTGGAG 3’  Rev 5’ TCTGCGATGATGATGATGTT 3’ |
| *Pcx* | IDT | Fwd 5’ GGAGCTAACATCTACCTTCTG 3’  Rev 5’ TAGGCTTATACTCCAGACGC 3’ |
| *Fbp1* | IDT | Fwd 5’ TGACCTGGTGATCAATATGC 3’  Rev 5’ CAAAAATGGTTCCGATGGAC 3’ |
| *Pck1* | IDT | Fwd 5’ TGAGATCTAGGAGAAAGCCA 3’  Rev 5’ CCTTGAAGTGGAACCAAAAC 3’ |
| *G6pc1* | IDT | Fwd 5’ CTAAAGCCTCTGAAACCCAT 3’  Rev 5’ ATGACTCAGTTTCCAGCATT 3’ |
| *Gk* | IDT | Fwd 5’ TCTTGAACCTGAGGATTTGT 3’  Rev 5’ TATGGGATACCACTTTCTGGA 3’ |
| *18s rRNA* | IDT | Fwd 5’ GCTTAATTTGACTCAACACGGGA 3’  Rev 5’ AGCTATCAATCTGTCAATCCTGTC 3’ |

**Supplementary Table 2: List of antibodies used for Western Blotting.**

| **Antibodies** | **Company** | **Catalog Number** |
| --- | --- | --- |
| Phospho-S6 Ribosomal Protein (Ser235/236) | Cell Signaling Technology | Cat# 4858S |
| Ribosomal Protein S6 Antibody (C-8) | Santa Cruz | Cat# sc-74459 |
| β-actin Monoclonal Antibody | Sigma | Cat# A5441 |
| Anti-rabbit IgG, HRP-linked Antibody | Cell Signaling Technology | Cat# 7074 |
| Anti-mouse IgG, HRP-linked Antibody | Cell Signaling Technology | Cat# 7076 |

**Supplemental Table 3: Circadian rhythm analysis of clock genes using JTK software.**

| **Gene** | **ADJ.P** | **PHASE** | **Circadian** |
| --- | --- | --- | --- |
| Bmal1 AL | 1.40E-06 | 22 | Yes |
| Bmal1 CR | 4.07E-07 | 20 | Yes |
| Bmal1 TR12 | 4.00E-08 | 0 | Yes |
| Per1 AL | 3.03E-06 | 10 | Yes |
| Per1 CR | 0.004539 | 10 | Yes |
| Per1 TR12 | 9.39E-07 | 14 | Yes |
| Per2 AL | 0.00026 | 14 | Yes |
| Per2 CR | 0.021337 | 14 | Yes |
| Per2 TR12 | 3.03E-06 | 16 | Yes |
| Rev erb a AL | 9.61E-14 | 6 | Yes |
| Rev erb a CR | 4.00E-08 | 6 | Yes |
| Rev erb a TR12 | 4.07E-07 | 10 | Yes |

**Supplemental Table 4: Circadian rhythm analysis of glucose metabolic genes using JTK software.**

| **Gene** | **ADJ.P** | **PHASE** | **Circadian** |
| --- | --- | --- | --- |
| Gck AL | 0.021337 | 16 | Yes |
| Gck CR | 0.001189 | 18 | Yes |
| Gck TR12 | 6.22E-07 | 18 | Yes |
| Pfk1 AL | 0.49115 | 6 | No |
| Pfk1 CR | 1 | 18 | No |
| Pfk1 TR12 | 0.014834 | 0 | Yes |
| Pcx AL | 0.185617 | 10 | No |
| Pcx CR | 1 | 10 | No |
| Pcx TR12 | 1 | 12 | No |
| Pck AL | 6.30E-06 | 10 | Yes |
| Pck CR | 0.000198 | 12 | Yes |
| Pck TR12 | 0.00034 | 12 | Yes |
| Fbp1 AL | 0.14151 | 10 | No |
| Fbp1 CR | 1 | 18 | No |
| Fbp1 TR12 | 1 | 6 | No |
| G6pc AL | 0.030246 | 12 | Yes |
| G6pc CR | 0.000935 | 16 | Yes |
| G6pc TR12 | 0.211662 | 6 | No |
| Gk AL | 0.092109 | 10 | No |
| Gk CR | 1 | 18 | No |
| Gk TR12 | 0.012299 | 10 | Yes |
